# Effects of a six-week basketball-specific individual skills development program on mental toughness and sport competition anxiety

**DOI:** 10.3389/fpsyg.2026.1794140

**Published:** 2026-05-11

**Authors:** Atakan Yazıcı, Ramazan Taşçıoğlu, Serdar Solmaz, İhsan Sari, Emirhan Kan, Nazmi Bayköse

**Affiliations:** 1 Dr. Performance Skills & Mindset, Amsterdam, Netherlands; 2 Amsterdam Basketball Academy, Amsterdam, Netherlands; 3 Department of Physical Education and Sports, Faculty of Sports Sciences, Ardahan University, Ardahan, Türkiye; 4 LFE Research Group, Department of Health and Human Performance, Faculty of Physical Activity and Sport Science (INEF), Universidad Politécnica de Madrid, Madrid, Spain; 5 Department of Sport Management, Faculty of Sport Sciences, Batman University, Batman, Türkiye; 6 Department of Physical Education and Sports, Faculty of Sports Sciences, Sakarya University of Applied Sciences, Sakarya, Türkiye; 7 Department of Physical Education and Sports, Faculty of Sports Sciences, Atatürk University, Erzurum, Türkiye; 8 Department of Coaching Education, Faculty of Sport Sciences, Akdeniz University, Antalya, Türkiye

**Keywords:** basketball, individual skills development, mental health, mental toughness, sport competition anxiety

## Abstract

**Introduction:**

This quasi-experimental study aimed to investigate the impact of a six-week basketball-specific individual skills development program on mental toughness and competitive anxiety among young professional male basketball players competing in organized leagues. Mental toughness and anxiety are critical psychological factors that influence athletic performance, particularly in high-pressure sports like basketball. The study sought to bridge the gap in existing research by examining how individualized training programs can simultaneously enhance mental resilience and reduce anxiety levels during the competitive season.

**Methods:**

Sixty-three male basketball players (*M* = 17.93, SD = 0.75), all of whom were officially registered players competing under club contracts and receiving financial compensation, were divided into experimental and control groups. The experimental group underwent a six-week tailored training program designed to enhance position-specific skills, while the control group participated in regular team training. Psychological assessments, including the Sports Mental Toughness Questionnaire (SMTQ-14) and the Sport Competition Anxiety Test (SCAT-A), were conducted before and after the intervention to measure changes in mental toughness and anxiety levels. Statistical analyses, including two-factor ANOVA and Mann–Whitney U tests, were used to evaluate the data.

**Results:**

The experimental group showed significant improvements in mental toughness subscales (confidence, consistency, and control) and a significant reduction in sport competition anxiety compared to the control group (*p* < 0.05). Effect sizes indicated moderate to large improvements in psychological outcomes, suggesting that the individualized skills development program effectively enhanced mental resilience and reduced anxiety among participants.

**Discussion:**

The findings highlight the importance of tailored training programs in fostering psychological resilience and reducing anxiety in basketball players. The results align with previous research emphasizing the role of structured, individualized training in improving mental toughness and managing competitive anxiety. The study underscores the need for coaches to integrate psychological skill development into training regimens, particularly during the competitive season, to optimize both performance and mental wellbeing.

**Conclusion:**

This study demonstrates that a six-week basketball-specific individual skills development program enhances players’ mental toughness and reduces sport competition anxiety. The findings highlight the effectiveness of personalized training programs in improving psychological performance, providing valuable insights for coaches and sports psychologists.

## 1 Introduction

Basketball is a multifaceted sport that requires the simultaneous development of psychological, physiological, and cognitive attributes to achieve optimal performance. Research has emphasized the impact of individual attributes, such as mental toughness and emotional intelligence, on team success, highlighting their critical roles in sustaining high-level performance (Carron and Eys, 2012; Cotterill and Fransen, 2016). Mental toughness, defined as an athlete’s ability to maintain optimal functioning under pressure, is particularly significant for basketball players who must navigate the combined aerobic and anaerobic demands of the sport (Mahoney et al., 2014; Mahoney et al., 2016). Mental toughness is a psychological asset that empowers athletes to begin and maintain goal-oriented efforts to reach peak performance, even when facing stressors of varying duration, frequency, and intensity (Stamatis et al., 2020).

Different conceptualizations of mental toughness in sport have been proposed by researchers. These conceptualizations are based on various perspectives (trait vs. state), components (such as self-beliefs, motivation, attitudes, coping and psychological skills, cognitions, and abilities), or the ways in which mental toughness impacts athletes’ behavior (e.g., rebounding after failures, maintaining consistency, effectively coping with pressure, and retaining focus despite distractions) (Hsieh et al., 2024). From one perspective, some studies indicate that mental toughness is a relatively stable psychological feature that enables athletes to confront and manage various types of pressure in order to achieve optimal performance (Gucciardi et al., 2009a; Middleton et al., 2004). However, from another perspective, mental toughness is also conceptualized as a state-like psychological resource that is purposeful, flexible, and efficient in supporting the achievement and maintenance of goal-directed pursuits. Accordingly, mental toughness may vary across situations or over time and is open to development or change (Gucciardi, 2017). Building on these perspectives, mental toughness and sport competition anxiety are theoretically interconnected. According to Clough et al.’s (2002) 4Cs model (Control, Commitment, Challenge, and Confidence), mental toughness helps athletes maintain emotional control, reappraise stressors as challenges, and sustain self-confidence under pressure. In line with this framework, these components directly influence the cognitive and somatic dimensions of competitive anxiety described in Martens et al. (1990) Multidimensional Anxiety Theory.

Consistent with this theoretical linkage, higher levels of mental toughness are associated with lower cognitive worry and somatic arousal, as well as more facilitative interpretations of anxiety symptoms. In this framework, mental toughness acts as a psychological buffer that reduces the debilitating effects of competitive anxiety and supports performance in high-pressure situations such as basketball competitions. Accordingly, advancing evidence-based mental toughness training practices requires a clearer understanding of effective strategies for promoting this construct. Therefore, implementing practices aimed at enhancing athletes’ mental toughness is of great importance (Stamatis et al., 2020). In addition to mental toughness, sport competition anxiety is another critical factor that affects athletes’ performance. Anxiety can impair decision-making, focus, and overall effectiveness in competitive settings (Judge et al., 2016; Javier Ponseti et al., 2016). In this regard, athletes often perceive anxiety as detrimental to their motor abilities, and it can disrupt sport performance (Weinberg and Gould, 2024).

The most widely accepted framework explaining competitive anxiety is the Multidimensional Anxiety Model proposed by Martens (1977), which includes cognitive anxiety, somatic anxiety, and self-confidence. Cognitive anxiety involves concerns and negative expectations regarding performance, whereas somatic anxiety is characterized by physical symptoms such as sweaty palms, muscle tension, and increased heart rate. Importantly, both excessively high and low levels of somatic anxiety may impair performance; elevated levels are associated with autonomic symptoms, while reduced levels are linked to fatigue, boredom, and lethargy. Thus, this model provides insight into the diverse symptoms of competitive anxiety experienced by athletes (Tóth et al., 2022). Building on this theoretical background, various psychological interventions have been utilized to assist athletes in managing competitive anxiety. Most studies have focused on mental skills techniques aimed at reducing anxiety intensity or promoting more facilitative interpretations of anxiety symptoms (Mellalieu et al., 2006). More recently, alternative approaches such as mindfulness (Noetel et al., 2019) and biofeedback training (Pusenjak et al., 2015) have also been explored.

Tailored training programs provide structured environments that not only develop technical skills but also promote psychological stability, reducing anxiety and improving confidence (Smoll et al., 2007; Smith et al., 2007). Individualized training programs have been shown to positively influence performance by addressing athletes’ specific needs, which traditional team practices may overlook (Bull et al., 2005; Gucciardi et al., 2009b). Yazıcı et al. (2021) reported that mental toughness and emotional intelligence differ significantly between Turkish and American professional basketball players, underlining the importance of culturally tailored training interventions. However, most studies have independently examined psychological or physiological aspects of performance, leaving the interaction between these domains underexplored (Duvan et al., 2010). Furthermore, previous research has primarily focused on general training approaches, with limited attention to position-specific, individualized basketball training programs and their psychological outcomes. In particular, the simultaneous effects of such training interventions on both mental toughness and sport competition anxiety during the competitive season remain largely unexplored. Recent studies have also highlighted that young professional athletes experience significant psychological challenges, such as the negative impact of high neuroticism on subjective wellbeing, which can be buffered by self-esteem and perceived social support (Solmaz, 2025).

Therefore, this study aims to address these gaps by simultaneously examining the effects of a basketball-specific, position-based, individualized skills development program on both mental toughness and sport competition anxiety. Unlike previous studies, this research focuses on the combined psychological outcomes of a structured individual training intervention implemented during the competitive season. In addition, the program is tailored according to playing positions, providing a more ecologically valid and sport-specific approach. By doing so, this study offers a more integrated understanding of how individualized training influences psychological performance in young professional basketball players. To the best of our knowledge, no previous study has examined these variables together within a position-specific individualized training framework in a real competitive-season setting.

Two hypotheses were set in this research: (i) Individual skills development program combined with basketball training will cause significantly greater changes in sport competition anxiety levels. (ii) An individual skills development program combined with basketball training will cause significantly greater changes in sports mental toughness. If an individual skills development program combined with basketball training improves players’ psychological performance more than basketball training alone does during similar training periods, basketball coaches will have evidence of more efficient training.

## 2 Materials and methods

### 2.1 Experimental design

This quasi-experimental study employed a pretest-posttest control group experimental design, a model commonly used to determine cause-and-effect relationships under the direct control of the researcher (Creswell and Creswell, 2017). As participants were not randomly assigned to groups, this design may be subject to potential threats to internal validity.

Before the experimental and control groups were formed, all participants completed a 2-week aerobic training program that included five low-intensity activities per week, 60–90 min each, to adapt to training and avoid injuries. This adaptation phase is a preferred approach in quasi-experimental research on team sports before the treatment (Yüksel et al., 2023). Following the adaptation phase, the Nonequivalent (Pretest and Posttest) Control-Group Design approach was used to assign control and experimental groups. In this design, while both groups take pre-and post-tests, only the experimental group receives the treatment, and the experimental and control groups are chosen without random assignment (Sheard et al., 2009).

**Figure 1:**
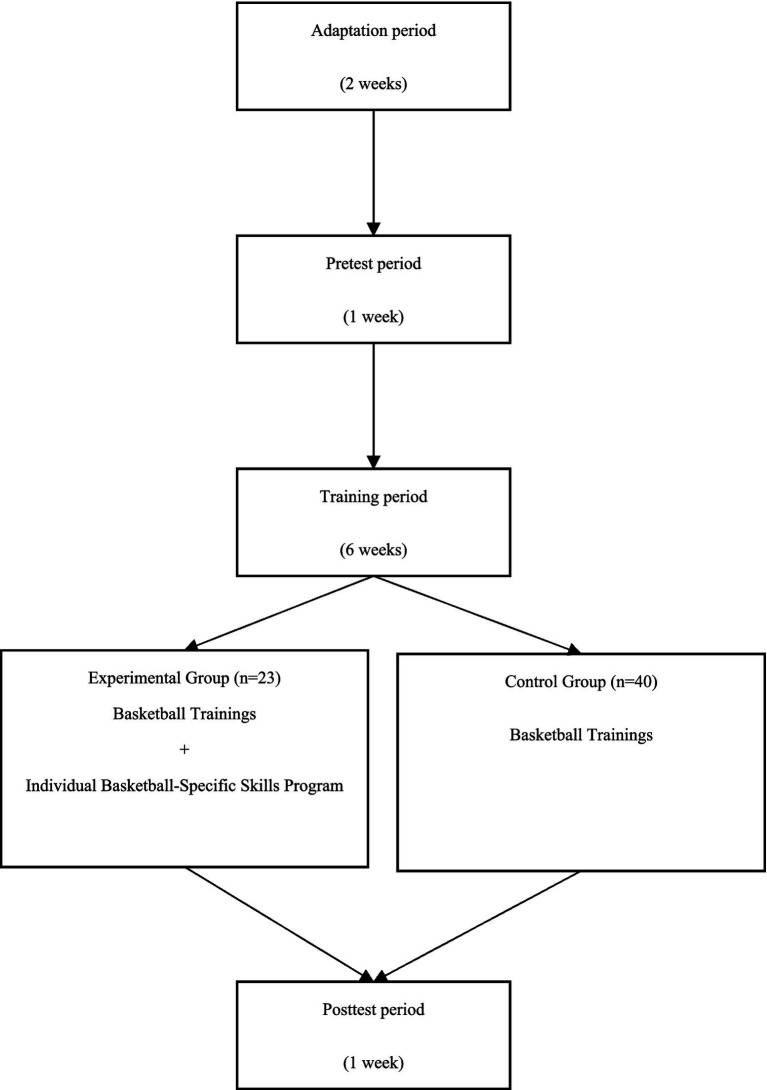
The research design.

After that, the experiment and control groups were engaged in the same team training program 5 days a week. Additionally, participants in the experimental group completed an individualized basketball-specific skills development program for 80 min, 3 days per week, over 6 weeks (Figure 1).

#### 2.1.1 Six-week basketball-specific individual skills development program

The experimental group athletes were divided into six subgroups based on their basketball positions, with no intermediate positions included. Players were categorized into three primary positions: guards (*n* = 11), forwards (*n* = 9), and centers (*n* = 3). Training plans were tailored to the positional requirements of each athlete and focused on developing position-specific skills (Table 1).

**Table 1:** Individual basketball-specific skills program.

Phase / Section	Content	Guards (G)	Forwards (FV)	Centers (C)
1	Warm up 15 min.	Box Drill (3 min. each) -Catch& one dribling pull up -Between /Between Pull up -Between spot up -Side forward dribling pull up -Side forward& snatch dribling pull up	Box Drill (3 min. each) -Catch& one dribling pull up -Between /Between Pull up -Between spot up -Side forward dribling pull up - Side forward& snatch dribling pull up	Box Drill (3 min. each) -Low pick up -High pick up -Power lay up inside hand -Pump fake & Step through -Spin & Step through
3	Main Programme 40 min	(1) Gilbert Drill (3 pts- 3 players in the each group) -Goal is to improve transition shot and spot up 2 min × 2 sets each players (15 min) (2) PNR situation against passive defence -Goal is to improve pull up skills in the 2 on 2 small sided games (A) Against drop defence (B) Against switch defence (C) Snake action (D) Jail situation (E) Boomerang action Total 2 min each situation in the both side of the wings(total = 20 min) (3) Free throw shooting as a group 10 makes in a row (5 min)	(1) Gilbert Drill (3 pts- 3 players in the each group) -Goal is improve transition shot and spot up 2 min × 2 sets each players (15 min) (2) PNR situation against passive defence -Goal is improve pull up skills in the 2 on 2 small sided games (A) Against drop defence (B) Against switch defence (C) Spanish action (D) Peja action (E) Boomerang action Total 2 min each sitation in the both side of the wings(total = 20 min) (3) Free throw as a group 10 makes in a row (5 min)	(1) Gilbert Drill (2 pts- 3 players in the each group) -Goal is to improve face up situation 2 min × 2 sets each players (15 min) (2) PNR situation against passive defence -Goal is to improve short & deep roll skills in the 2 on 2 small sided games (A) Slip Screen (short) (B) Pop-out (C) Re-screen (short) (D) Angle Screen(deep) (E) HO&re-screen (deep) Total 2 min each sitation in the both side of the wings(total = 20 min) (3) Free throw as a group 10 makes in a row (5 min)
4	Cool Down 15 min.	5 Spot 10/14 Static Shot (3pts)	5 Spot 10/14 Static Shot (3pts)	5 Spot 10/14 Static Shot (2pts)

G, Guards; FV, Forwards; C, Centers.

### 2.2 Participants

The participants were 63 young professional (scholarship) male basketball players actively competing (Mean_Age_ = 17.93 ± 0.75) from the private high schools sports clubs and the high school’s basketball team during the 2019–2020 season in Eskişehir, Türkiye. Participants were divided into an experimental group (*n* = 23, Mean_Age_ = 18.08 ± 0.79) and a control group (*n* = 40, Mean_Age_ = 17.85 ± 0.73).

All participants were officially licensed basketball players competing in organized leagues under formal club contracts. Despite their relatively young age, they were considered professional athletes as they received financial compensation and regularly participated in competitive league matches.

### 2.3 Quantitative measures

#### 2.3.1 Sport mental toughness questionnaire (SMTQ)

The Sport Mental Toughness Questionnaire (SMTQ) was developed by Sheard et al. (2009) to assess mental toughness levels in sports environments. The Turkish adaptation of the SMTQ was conducted by Altıntaş and Bayar (2016). The instrument comprises 14 items and three subscales: confidence, consistency, and control. McDonald’s omega coefficients for the SMTQ subscales, as presented in Table 2, range from 0.680 to 0.789, indicating acceptable reliability for measuring mental toughness in sports settings (Altıntaş and Bayar, 2016). In addition, previous studies have supported the construct validity of the SMTQ, demonstrating its suitability for assessing mental toughness across different sport contexts (Sheard et al., 2009; Altıntaş and Bayar, 2016).

**Table 2:** The McDonald’s omega reliability coefficients.

Variables	The McDonald’s omega reliability coefficients
SCAT-A	0.925
Confident	0.786
Consistency	0.789
Control	0.680

#### 2.3.2 Sport competition anxiety test (SCAT-A)

The Sport Competition Anxiety Test (SCAT-A) was originally developed by Martens (1977) and later adapted into Turkish by Koruç (1998). The SCAT-A consists of 15 items formatted as a three-point Likert scale. Of these, 10 items measure individual differences in competition-related trait anxiety, while the remaining five serve as filler questions and are not scored. The original scale demonstrated high internal consistency, with KR-20 values ranging from 0.95 to 0.97. In the present study, the McDonald’s omega reliability coefficient for SCAT-A was found to be 0.925 (see Table 2), indicating high internal consistency. Furthermore, the SCAT-A has demonstrated satisfactory construct and criterion-related validity in both the original and Turkish adaptation studies, supporting its use for assessing competitive anxiety in sport settings (Martens, 1977; Koruç, 1998).

### 2.4 Data analysis

The research used two-factor ANOVA to evaluate whether there was a statistically significant difference in the two groups’ pre-test and post-test scores (Pallant, 2011). Furthermore, a Mann–Whitney U test was conducted to assess the homogeneity between the experimental and control groups. No significant differences were found between the groups in terms of initial baseline characteristics. This analytical approach ensures a robust and accurate interpretation of the intervention’s effects while adhering to statistical rigor. Cohen’s (Cohen, 1988) partial eta squared (*η*^2^) effect sizes and thresholds (0.00, 0.06, and.14 for small, medium, and large effect sizes, respectively) were used to compare differences between the groups. The McDonald’s omega reliability coefficients ranged from 0.680 to 0.925, indicating acceptable reliability for the variables (Table 2). SPSS 26 was used for the analysis. A significance level of *p* < 0.05 was adopted for all tests.

## 3 Results

The experimental group had greater changes in sport competition anxiety (F1,61 = 32.845, *p* = 0.000, η^2^ = 0.350), confidence (F1,61 = 57.953, *p* = 0.000, *η*^2^ = 0.487), consistency (F1,61 = 33.890, p = 0.000, η^2^ = 0.357), and control (F1,61 = 45.084, *p* = 0.000, *η*^2^ = 0.425) compared to the control group (Table 3).

**Table 3:** Means and standard deviations for sport competition anxiety and mental toughness.

Variables	Groups	Pretests	Posttests	*F*	*p*	η^2^
Sport Competition Anxiety	EG (*n* = 23)	23.60 ± 4.08	13.20 ± 1,24	G = 53.449	0.000	0.985
	CG (*n* = 40)	23.95 ± 3.82	22.33 ± 4.13			
				T = 61.751	0.000	0.503
				GxT = 32.845	0.000	0.350
Confidence	EG (*n* = 23)	15.75 ± 2.00	22.15 ± 2.78	G = 93.080	0.000	0.604
	CG (*n* = 40)	15.07 ± 1.99	14.05 ± 3.09			
				T = 30.404	0.000	0.333
				GxT = 57.953	0.000	0.487
Consistency	EG (*n* = 23)	10.55 ± 1.05	14.45 ± 1.67	G = 83.735	0.000	0.579
	CG (*n* = 40)	9.67 ± 1.57	9.33 ± 2.31			
				T = 23.674	0.000	0.280
				GxT = 33.890	0.000	0.357
Control	EG (*n* = 23)	9.75 ± 0.0.91	13.80 ± 1.79	G = 52.986	0.000	0.465
	CG (*n* = 40)	9.35 ± 1.21	9.35 ± 2.17			
				T = 45.084	0.000	0.425
				GxT = 45.084	0.000	0.425

EG, Experimental Group; CG, Control Group.

## 4 Discussion

This research aimed to investigate the effect of a six-week basketball-specific individual skills development program on mental toughness and sport competition anxiety. Two hypotheses were set for this purpose. Both hypotheses regarding mental toughness and sport competition anxiety were supported, indicating significant differences between experimental and control groups. In other words, there are two main findings of this research. One is that the experimental group enhanced their mental toughness with the individual skills development program compared to the control group. The other is that the experimental group reduced their sports competitive anxiety scores with the individual skills development program compared to the control group. Mental toughness, a critical psychological trait for optimal athletic performance, encompasses cognitive, emotional, and behavioral components that enable athletes to function effectively under stress (Mahoney et al., 2016). Hardy et al. (2014) further highlight that mental toughness is a multifaceted skill that integrates resilience, consistency, and emotional regulation in high-pressure environments. The significant improvements observed in the experimental group’s mental toughness scores in this study reinforce the importance of targeted individual development training for developing this crucial attribute and making players stronger in training and games. Moreover, Mamassis and Doganis (2004) have emphasized the detrimental effects of excessive sport competition anxiety on performance, which often leads to unfavorable outcomes, such as missed shots, turnovers, and poor decision-making. The significant reduction in anxiety levels among the experimental group participants underscores the efficacy of the individualized skills program in mitigating the debilitating effects of anxiety, thereby allowing players to perform with greater composure and confidence. Systematically planned and implemented individual development training enhances athletes’ belief in their own strengths. Coaches should design comprehensive and long-term individual development plans that include components and game tactics appropriate to the athlete’s capacity and implement them within the team organization. This approach plays a significant role in enabling athletes with limited athletic abilities to potentially reach championship levels.

The observed decrease in sport competition anxiety for the experimental group mirrors the findings of numerous studies examining the effects of various mental training interventions on anxiety reduction in athletes (Gould et al., 2002; Kendall et al., 1990; González-Hernández et al., 2020). For instance, Nicholls et al. (2007) found that toughness resources, such as self-confidence and the ability to cope with and control anxiety, are positively correlated with athletic performance across different team sports.

Research on developing mental toughness emphasizes experiential learning and structured training. Thelwell et al. (2010) found that mental toughness can be cultivated through consistent practice, goal-setting, and competitive simulation. Athletes in their study reported that well-designed training programs significantly contributed to their mental toughness. Similarly, Bull et al. (2005) indicated that athletes’ belief in the quality of their training directly influences their mental toughness. This aligns with our study’s findings, where individualized training enhanced athletes’ confidence and consistency. Gucciardi et al. (2009b) explored the role of coaching philosophies and challenging experiences in fostering mental toughness, noting that athlete-coach relationships and tailored training regimens are pivotal in this process.

The environmental and interpersonal context of training also plays a role. Mahoney et al. (2014) demonstrated that supportive social environments and exposure to challenging yet manageable tasks significantly improve athletes’ mental toughness. Additionally, Beattie et al. (2019) emphasized that the quality of training, rather than its intensity, serves as a key determinant of mental toughness development. In light of these studies, our findings support the integration of structured and position-specific training programs to optimize psychological outcomes in basketball players.

Anxiety, the second variable examined, was also significantly reduced among the experimental group participants. Anxiety has long been recognized as a determinant of athletic performance, influencing focus, decision-making, and execution under competitive conditions (Judge et al., 2016; Javier Ponseti et al., 2016; Mabweazara et al., 2017). Several interventions have been explored to mitigate anxiety in athletes. For example, Smoll et al. (2007) conducted training programs for athletes’ parents and coaches, creating supportive environments that reduced anxiety. Similarly, Smith et al. (2007) highlighted the importance of motivational climate interventions for coaches in alleviating competitive anxiety among athletes.

Visualization and mental training have also proven effective. Williams and Cumming (2016) identified imagery techniques as valuable tools for managing competitive anxiety, while Fortes et al. (2016) demonstrated the anxiety-reducing effects of mental training in swimmers. Our study aligns with these findings, showing that basketball-specific individual training programs not only address physiological performance but also contribute to psychological stability by reducing anxiety levels.

The impact of physical activity on anxiety is well-documented. Aerobic and resistance training interventions have consistently shown positive effects on athletes’ situational anxiety (Hale et al., 2002). For example, Fabre et al. (2002) compared the cognitive and anxiety-reducing effects of aerobic and mental training, finding that combined interventions yielded superior outcomes in memory performance and anxiety reduction. Rashidi et al. (2017) reported significant reductions in depression and anxiety following an eight-week aerobic exercise program, while Budde et al. (2016) observed similar effects among children participating in a ten-week aerobic training regimen.

Previous research found that elite male athletes at different performance levels have similar baseline mental endurance levels (Yarayan et al., 2024). The current findings indicate that individual skill training can effectively enhance mental toughness and endurance across performance levels. As a result, our findings contribute to this growing body of evidence by demonstrating that individualized skills-based training programs in basketball reduce anxiety while improving critical psychological attributes such as mental toughness. These interventions not only enhance performance but also support athletes’ overall wellbeing, underscoring their value in comprehensive sports training programs. Although the mean age of the participants was relatively low, all players were actively competing in organized leagues under formal contracts and receiving financial compensation. Therefore, they can be classified as young professional athletes within the context of this study.

Despite the promising results, this study has several limitations that should be considered when generalizing findings and applying practical recommendations. First, the non-randomized design may introduce potential threats to internal validity, such as selection bias and maturation effects, although baseline equivalence was confirmed. Second, the sample was limited to adolescent male players from a single club, which may restrict the generalizability of findings to other populations, including female athletes or players from different cultural contexts. Third, the intervention lasted only 6 weeks, and the long-term effects on mental toughness and competition anxiety remain unknown. Finally, data were collected via self-report questionnaires, which may be subject to response biases.

## 5 Conclusion

The findings of this study highlight the effectiveness of a six-week individualized skills development program in enhancing basketball players’ mental toughness and reducing sport competition anxiety. Statistical analyses revealed significant differences between the experimental and control groups across mental toughness subscales and total anxiety scores. Participants in the experimental group exhibited substantial improvements in cognitive, emotional, and behavioral aspects of mental toughness, including confidence, persistence, and control. Furthermore, their anxiety levels decreased significantly, suggesting that the individualized training program mitigated the negative effects of competition-induced stress. These results reinforce the value of personalized training interventions in fostering psychological resilience and improving performance outcomes in basketball players.

### 5.1 Practical implications

From an expert perspective, coaches and sports organizations, from professional to youth levels, may consider prioritizing the implementation of individualized skills development training programs during the season. The success of such programs heavily relies on the expertise and leadership skills of the head coach, whose ability to design comprehensive and strategic training plans tailored to the athlete’s specific needs is paramount. In this context, the head coach must possess a deep understanding of both technical and psychological development to ensure that the training program holistically addresses the athlete’s performance requirements.

In addition, the role of individual skill development coaches (head or assistant coach) has become increasingly prevalent in modern basketball, but concerns remain regarding their qualifications and the objective standardization of their training methods. Literature suggests that individual skill development coaches may vary in their certification levels and the use of evidence-based frameworks, which can result in potential inconsistencies in the content and philosophy of training programs. Training programs that are less structured or lack depth may not fully address the complexities of skill acquisition, which could influence athletes’ technical development and mental toughness.

To mitigate these risks, establishing regulatory frameworks and certification processes for skills coaches could be beneficial, helping ensure that they are equipped with appropriate knowledge and tools to provide structured training. For example, while the Turkish Basketball Federation mandates a minimum Level 4 coaching certification, higher-level coaching may provide additional benefits for individual skills development. Participation of Level 5 (A Category) coaches in examination programs could enhance training quality, and periodic re-evaluation of experienced coaches may help them stay updated on contemporary approaches. Such measures, if implemented, may help create an environment where athletes benefit from well-rounded, scientifically grounded, and strategically designed training programs.

## Data Availability

The original contributions presented in the study are included in the article/Supplementary material, further inquiries can be directed to the corresponding author.
