# Supplementary material for: Effects of a six-week basketball-specific individual skills development program on mental toughness and sport competition anxiety
Source: Front Psychol. 2026 May 11;17:1794140. doi: 10.3389/fpsyg.2026.1794140 (PMC13198994; doi:10.3389/fpsyg.2026.1794140)
Supplement: Supplementary file 1 [file Table_1.DOCX]

The criticism regarding the Cronbach's alpha coefficient in the previous review has been taken into account. An evaluation and re-analysis process has been carried out on this matter. In the evaluation, the small number of items and the limited sample size of the scale are considered factors that reduce the alpha coefficient. However, reliability coefficients were re-analyzed with McDonald's Omega, and the results showed sufficient internal consistency coefficients. Moreover, the use of the scale in the literature was rechecked. Therefore, it is thought that the McDonald's Omega internal consistency coefficients and the scale's popularity in the literature were powerful evidence for the reliability of the scales. In other words, the reliability concerns of the scale in the previous review have been addressed in this version by strengthening them with studies in the literature. Furthermore, Table 1, which provides sufficient detail about the specific nature of the intervention program, has been presented in detail. This table provides sufficient information for the repetition of the study in future research. The long-term effects of the results are not within the scope of this study. The findings of the study focus on effects during the season. Thus, it was expressed in the abstract, "the findings suggest that implementing individualized skills development programs during the season can significantly reduce competitive anxiety levels and enhance mental toughness.
